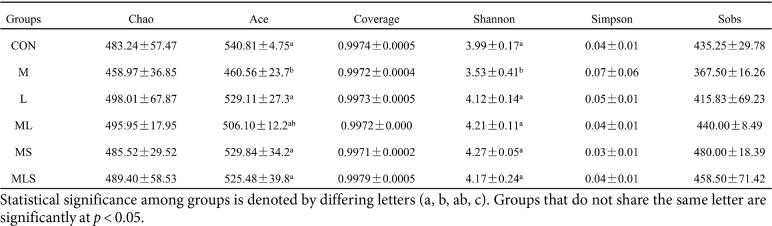# Potential Protective Effect of Selenium-Enriched *Lactobacillus plantarum* on Cadmium-Induced Liver Injury in Mice

**DOI:** 10.4014/jmb.2312.12051

**Published:** 2024-04-23

**Authors:** Yanyan Song, Jing Zhang, Yidan Li, Yuxuan Wang, Yingxin Wan

**Affiliations:** College of Biochemical Engineering, Beijing Union University, Beijing 100023, P.R. China

**Keywords:** Selenium-enriched *Lactobacillus plantarum*, selenium nanoparticles, liver, Cd

## Abstract

Cadmium (Cd) is a prevalent environmental contaminant that poses a potential hazard to the health of both humans and animals. In this study, biosynthesized selenium-enriched *Lactobacillus plantarum* and selenium nanoparticles (SeNPs) were developed and evaluated for their protective effects against Cd-induced hepatic injury in mice through oral administration for 4 weeks. Cadmium exposure resulted in severe impairment of liver function, as evidenced by increased levels of serum markers of liver injury and, oxidative stress and significant damage to liver tissue, and a notable decrease in the diversity of the intestinal microbiota. Oral administration of Se-enriched *L. plantarum* (LS) reduced cadmium accumulation in the liver by 49.5% and, restored other cadmium-induced damage markers to normal levels. A comparison of the effects with those of *L. plantarum* (L) and SeNPs isolated from LS revealed that LS could more effectively alleviate hepatic oxidative stress and reduce the intrahepatic inflammatory responses of the liver, further protecting against cadmium-induced liver injury. These findings suggest that the development of LS may be effective at protecting the liver and intestinal tract from cadmium-induced damage.

## Introduction

Probiotics such as *Lactobacillus* and *Bifidobacterium*, are known to regulate intestinal function, enhance immunity, and lower cholesterol levels [1-3]. In recent years, probiotics have been shown to have additional functions, such as reducing the toxicity of heavy metals by adsorbing, transforming, and dissolving them [4]. Previous studies have shown that *Lactobacillus plantarum* CNR273 can normalize antioxidant parameters in the mouse liver, regulate the intestinal flora, and decrease cadmium (Cd) accumulation in tissues [5]. The importance of probiotics in injury has been strongly suggested because probiotics induce sirtuin1-activated peroxisome proliferator-activated receptor gamma coactivator-1α (PGC-1α), which in turn, attenuates hepatic mitochondrial damage, mitochondrial swelling, and hepatic tissue necrosis in mice [6]. In a rat model of hepatic fibrosis, administration of the probiotic *Lactobacillus* attenuated oxidative stress, inflammation and fibrosis in the mouse liver [7]. Moreover, probiotics administration resulted in a significant increase in the relative abundance of *Faecalibacterium prausnitzii* and *Prevotella* in feces compared to their abundance in the control group, whereas there was no significant difference in α- or β-diversity [8]. *F. prausnitzii* has an anti-inflammatory effect, and *Prevotella* is the cornerstone genus in the gut. Therefore, probiotics play an important role in attenuating liver injury, maintaining host-gut microbial homeostasis, and mitigating intestinal injury.

Selenium (Se), an essential trace element, plays an important role in the treatment of cancer, heavy metal poisoning and other diseases [9, 10]. However, traditional Se supplements are highly toxic. To alleviate heavy metal toxicity, cancer and kidney disease, nano Se and organic Se are widely used due to their lower toxicity and higher bioavailability than inorganic Se. On one hand, Se can balance the oxidation-reduction state in the body, protect cells from oxidative damage, and maintain normal cell function. On the other hand, it can resist damage caused by many heavy metals [11]. As an important antioxidant, Se regulates the expression of selenoprotein-encoding genes, such as glutathione peroxidase (*GPX*) and thioredoxin reductase (*TrxR*) [12], making it effective at mitigating liver injury. Previous studies have increasingly shown that Se can reduce liver coefficient values in Cd-exposed mice by antagonizing the heavy metal Cd, increasing the activity of antioxidant enzymes, and increasing the ability to scavenge radicals [13].

Cd is a highly toxic heavy non-ferrous metal commonly used in alloys, anticorrosive coatings, pigments, radiation shielding, and semiconductors for solar cells [14]. Its use presents significant environmental pollution and health hazards globally. For instance, Cd in soil and water can be absorbed by certain crops and aquatic organisms, thus becoming enriched in the food chain [15], ultimately posing a threat to human life and health at the highest point of the food chain. The acute toxic effects of varying forms of Cd differ [16]. Cd-metallothionein is primarily stored in the kidneys, whereas Cd sulphate and inorganic Cd primarily accumulate in the liver, causing liver dysfunction [17]. Furthermore, Cd exposure of the gut microbiota may result in elevated lipopolysaccharide (LPS) production, impacting the metabolic activities of the gut microbiome. Heightened LPS production, can also impair barrier function, leading to endotoxemia and systemic inflammation [18]. Elafify *et al*. [19] found that, *Lactobacillus rhamnosus* can reduce the concentration of Cd in soft cheese by 74.5% at 4°C. Hower, lactic acid bacteria (LAB) are safe, efficient, and economical microorganism that can bind to Cd through chelation, complexation, and the formation of extracellular polymers, thus removing Cd and mitigating Cd-induced damage.

Based on the above mentioned studies on Se and probiotics, we hypothesized that the combination of Se and probiotics may have greater potential to mitigate Cd toxicity than Se or probiotics alone. Many LAB can convert inorganic Se into organic Se and nano-Se to form Se-enriched LAB with antioxidant activity [20, 21]. Additionally, synthetic Se-enriched lactobacilli have shown potential in repairing heavy-metal-induced damage [22, 23]. To explore whether the combination of Se and probiotics can mitigate Cd toxicity, we utilized Se-enriched *L. plantarum*, using a strain isolated from fermented sauerkraut from a Se-enriched region and, co-cultured with sodium selenite. Mice were administered Se-enriched *L. plantarum* for 4 weeks to evaluate its effects on Cd toxicity. Oral administration of *Lactobacillus* and Se nanoparticles (SeNPs) isolated from Se-enriched *L. plantarum* were also used to compare the effects of different treatments. Furthermore, changes in physiological status, liver function, and the intestinal microbiota of mice were analyzed after different treatments. This study is significant, as it elucidated that the combined application of Se and probiotics enhanced the anti-Cd toxicity ability of mice, providing a safe and effective method to alleviate Cd toxicity.

## Materials and Methods

### Bacterial Strain

*L. plantarum*, isolated from sauerkraut in Ankang, Shaanxi, was identified and preserved in the China General Microbiological Culture Collection Center (China).

### Preparation and Characterization of Se-Enriched *L. plantarum*

*L. plantarum* was cultured at 2% inoculum in De Man–Rogosa–Sharpe liquid medium at 37°C for 6 h. Subsequently, 4.0 mM sodium selenite was added to the medium, and the culture was continuously incubated at 37°C for another 24 h, resulting in the production of red-colored *L. plantarum* containing SeNPs. At the end of the incubation period, part of the culture was centrifuged (4,500 ×*g*, 10 min) and the precipitate was collected. The precipitate was rinsed three times with sterile water, frozen overnight at -80°C, and then lyophilized to obtain Se-enriched *L. plantarum*. In another part, biosynthesized SeNPs were isolated using the method reported by Xu *et al*.[24]. The lyophilized substances were then separately characterized and prepared as cell suspensions in distilled water for animal experiments.

The morphology of *L. plantarum* was characterized using scanning electron microscopy (SEM; Regulus 8230; Hitachi, Japan). The morphological properties of Se-enriched *L. plantarum* and the SeNPs were observed using transmission electron microscopy (TEM; JEM F200, Jeol, Japan) and energy-dispersive X-ray spectroscopy (EDS; Oxford Instruments, Oxford, England). The elemental compositions were analyzed using X-ray photoelectron spectroscopy (XPS; Thermo ESCALAB 250XI; Thermo Fisher Scientific, USA), and the crystalline form of SeNPs was analyzed using X-ray diffraction (XRD; D8; Bruker, Germany). Ethanol was added to the sample to dissolve the dispersion with sonication for 5 min. The sample was, then dripped onto a copper mesh and baked under a lamp until the solvent evaporated and then tested using TEM and EDS. SEM and EDS tests were performed by adhering the sample to a conductive adhesive. The sample was fixed to the sample stage with conductive adhesive and tested using XPS. A sample with a particle size of approximately 45 microns was spread over the entire stage for testing via XRD.

### Experimental Design and Sample Collection

Seventy-two 6-week-old male C57BL/6 mice were purchased from SPF Biotechnology Co, Ltd. (China). The animals were housed in a specific-pathogen-free facility for 1 week to acclimate before commencing treatment. Different agents were orally administered for 4 weeks. During the experimental period, the mice were housed in individual cages at 25°C, with 30%-70% humidity and a 12-h light/dark cycle. The 72 mice were randomly divided into six groups: the control (CON) group, the model (M) group, the *L. plantarum* (L) group, the model *L. plantarum* (ML) group, the model-SeNPs (MS) group, and the model-Se-enriched *L. plantarum* (MLS) group. The M group received an intraperitoneal injection of 2 mg/kg body weight (BW) CdCl_2_. The number of live bacteria was 1 × 10^8^ CFU/ml. Each mouse was force-fed 10 ml/kg BW *L. plantarum* or Se-enriched *L. plantarum*.

The body weight and dietary consumption of the mice were recorded every 2 days. After 4 weeks, feces were collected and, all mice were fasted overnight and subjected to isoflurane anesthesia. Blood was collected from the eyeball, and the mice were then sacrificed, and liver tissue was collected and stored at -80°C for subsequent testing.

### Blood Analysis

The blood samples were centrifuged at 3,000 rpm for 10 min at 4°C to separate and collect the serum, which was analyzed using a fully automated biochemical analyzer (Au480, Beckman USA). The serum levels of alanine aminotransferase (ALT), aspartate aminotransferase (AST), alkaline phosphatase (ALP), total cholesterol (TC), triglycerides (TG), high-density lipoprotein (HDL) and low-density lipoprotein (LDL) were measured.

### Histological Analysis of the Liver

Liver tissue samples were fixed with 4% paraformaldehyde, then trimmed, dehydrated, embedded, sectioned, stained, and sealed in strict accordance with the procedure for histopathological testing. Finally, qualified samples were examined microscopically using a slide scanner (PANNORAMIC DESK/MIDI/250/1000; 3DHISTECH, Hungary). Scanning software (CaseViewer 2.4, 3DHISTECH) was used to analyze the basic pathological changes.

### Measurement of Cd Concentrations

The liver tissue was transferred to polytetrafluoroethylene and, 4 ml of HNO_3_ and 1 ml of H_2_O_2_ were added. The obtained mixture was digested in a microwave digester (MARS, CEM, USA). After digestion, the samples were evaporated on a hot plate until almost dry. The remaining liquid was transferred to a 50 ml volumetric flask, to which 0.2 g of thiourea-ascorbic acid solution and 0.1 mg of cobalt solution were added. The Cd content was detected using an atomic fluorescence spectrometer (AFS-230E, Beijing Haiguang Co., China).

### Determination of Oxidative Stress Index

Oxidative damage was evaluated by measuring relevant oxidative stress indices in liver tissue. The kits purchased from Nanjing Jianjian Biological Research Institute (China) were used for the determination of superoxide dismutase (SOD), malondialdehyde (MDA), catalase (CAT) and glutathione peroxidase (GPX) levels.

### Analysis of the Gut Microbiota

To analyze the gut microbial composition, extracted genomic DNA was detected by 1% agarose gel electrophoresis assay, and the V3-V4 region of the bacterial 16Sr RNA gene was amplified. The forward primer used was 338F (5'-ACTCCTACGGGGAGGCAGCAG-3') and the reverse primer was 806R (5'-GGACTACHVGGGGTWTCTAAT-3'). PCR products were detected and quantified using the QuantiFluor-ST blue fluorescence quantification system (Promega, China). Sequencing libraries were constructed by using the Illumina MiSeq platform. The sequence data were analyzed by using the QIIME and R software packages (R Project for Statistical Computing, Austria). The α-diversity and β-diversity of the gut microbiota were analyzed at the level of amplicon sequence variation using principal coordinate analysis (PCoA).

### Data Analysis

All data were analyzed by one-way analysis of variance using SPSS (IBM, USA) followed by Tukey’s test. All the figures were constructed using Origin software (OriginLab., USA).

## Results

### Characterization of Se-Enriched *L. plantarum* and SeNPs

**Fig. 1:**
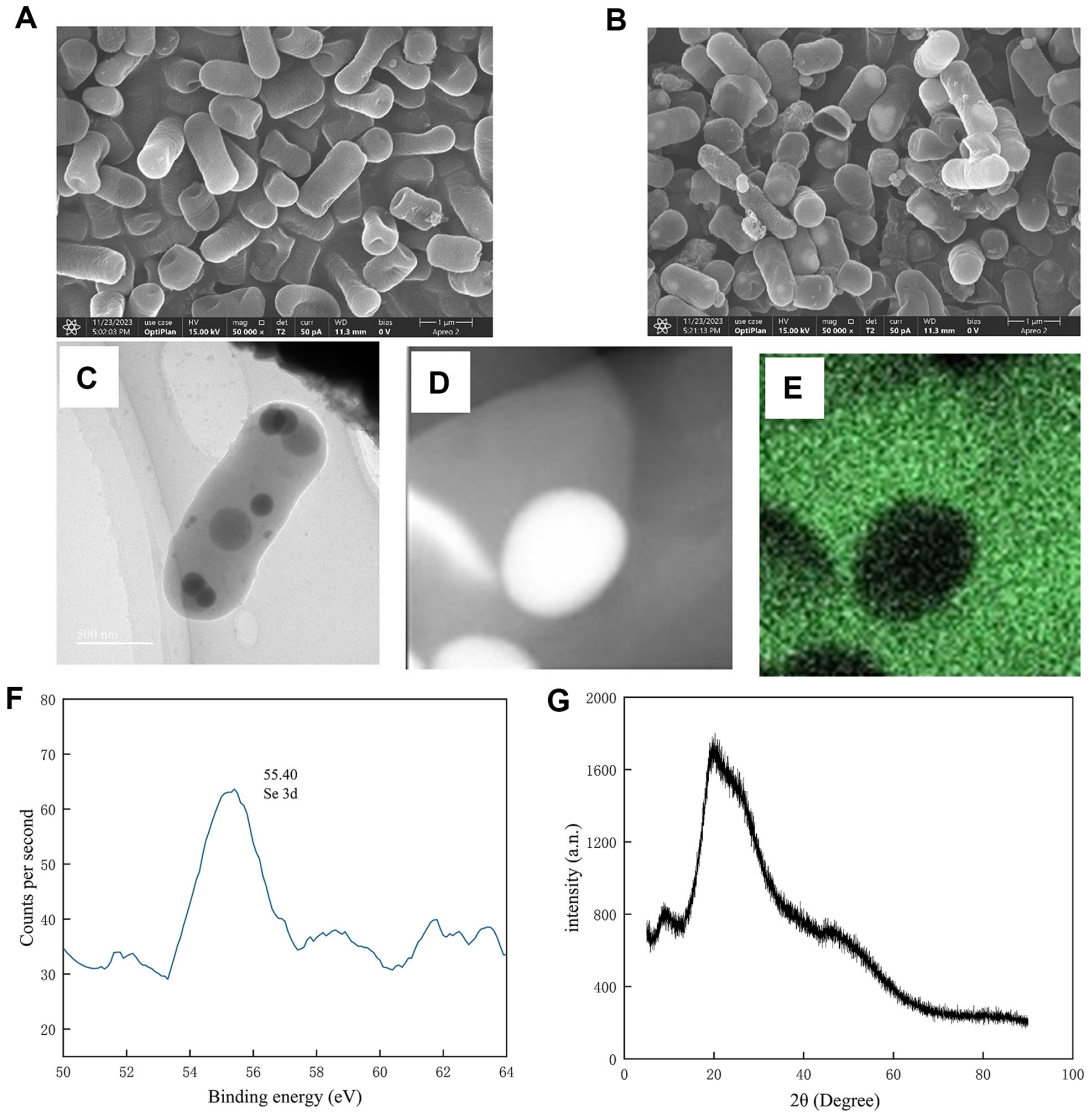
Characteristics of the prepared *L. plantarum* (**L**), Se-enriched *L. plantarum* (**LS**) and SeNPs: (**A**) SEM of L; (**B**) SEM and (**C**)TEM of LS; (**D**) TEM, (**E**) EDS, (**F**) XPS, and (**G**) XRD analyses of SeNPs.

SEM analysis was performed to determine the morphology of Se-enriched *L. plantarum* cells. The analysis showed no significant difference in the morphology of *L. plantarum* cells with (Fig. 1A) and without (Fig. 1B) Se enrichment. TEM analysis was performed to determine the location of the biosynthesized NPs produced by *L. plantarum*. The micrographs revealed that the NPs were deposited inside the cell (Fig. 1C), indicating the formation of SeNPs. Previous studies have shown that microorganisms can reduce selenite by the following mechanism. Glutathione (GSH) in the bacterial cell mediates the conversion of bisulfite to GSH-Se, which is further reduced to insoluble GSH-Se particles by GSH reductase, and finally reducible GSH and Se are formed [25]. Meanwhile, isolated SeNPs were morphologically intact (Fig. 1D and 1E). SeNPs with sizes from 5 nm to 200 nm have been reported to have significant biological function [26]. Therefore, the SeNPs formed in this study may also have high biological activity. In addition, the energy spectra of the SeNPs produced by *L. plantarum* were observed using XPS. The results showed the SeNPs comprised zero-valent elemental selenium (Fig. 1F). The EDX analysis showed that the SeNPs had an amorphous structure (Fig. 1G).

### Changes in Food Intake, Body Weight, and Liver Weight

The mice in the CON and L groups exhibited normal eating and drinking habits, had smooth and neat fur, and were in good spirits. In contrast, the mice in the M group had reduced feeding, were emaciated, had dull fur, and were in poor spirits. No significant differences were observed between the other treatment groups and the control group.

**Fig. 2:**
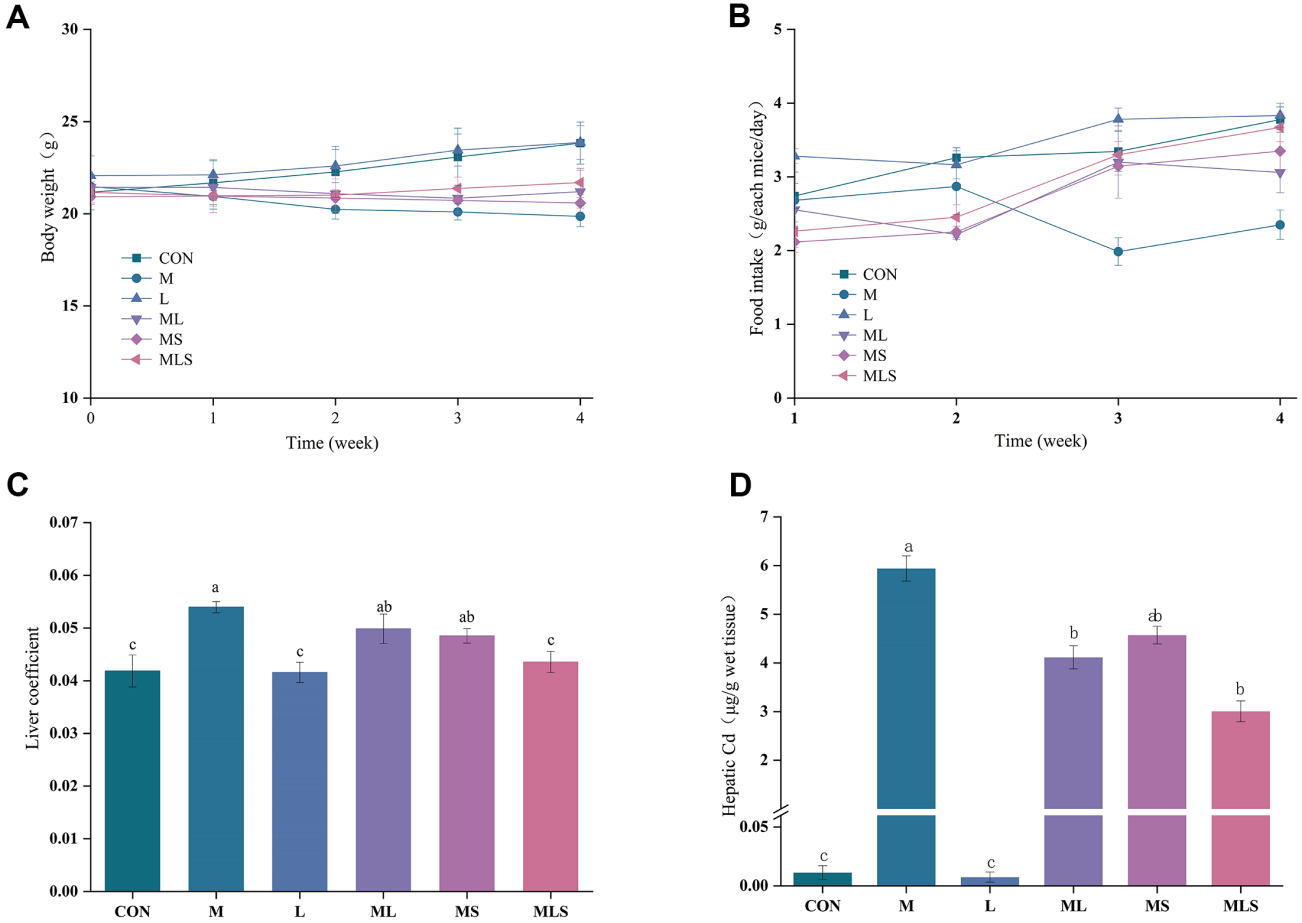
General characteristics and Cd content of mice. (**A**) Food intake, (**B**) Body weight, (**C**) Liver coefficient, (**D**) Cd content in the liver. Statistical significance among groups is denoted by differing letters (a, b, ab, c). Groups that do not share the same letter are significantly at *p* < 0.05.

As shown in Fig. 2A, 2B, 2C, mice exposed to Cd for 4 weeks had reduced food intake, decreased body weight, and increased liver weight, indicating that Cd was toxic. The treatment administered to the MLS group restored these indices to near-normal levels, as compared to the CON group (*p* < 0.05). The effects observed in the ML and MS groups were significantly less than those of the MLS group (*p* < 0.05).

### Cd Accumulation in the Liver

As shown in Fig. 2D, the levels of Cd deposition in the liver were determined to better explore the toxicity of Cd to the liver. The results confirmed that after Cd treatment, the model group had obvious Cd accumulation. Compared with the model group, Cd accumulation was significantly decreased in the ML, MS, and MLS groups (*p* < 0.05). The Cd content in the liver of the ML, MS and MLS groups was reduced by 30.8%, 23.0%, and 49.5%, respectively, showing that Se-enriched *L. plantarum* treatment was more effective at reducing Cd accumulation in the liver than the other treatments.

### Serum Levels of Markers of Liver Function

**Fig. 3:**
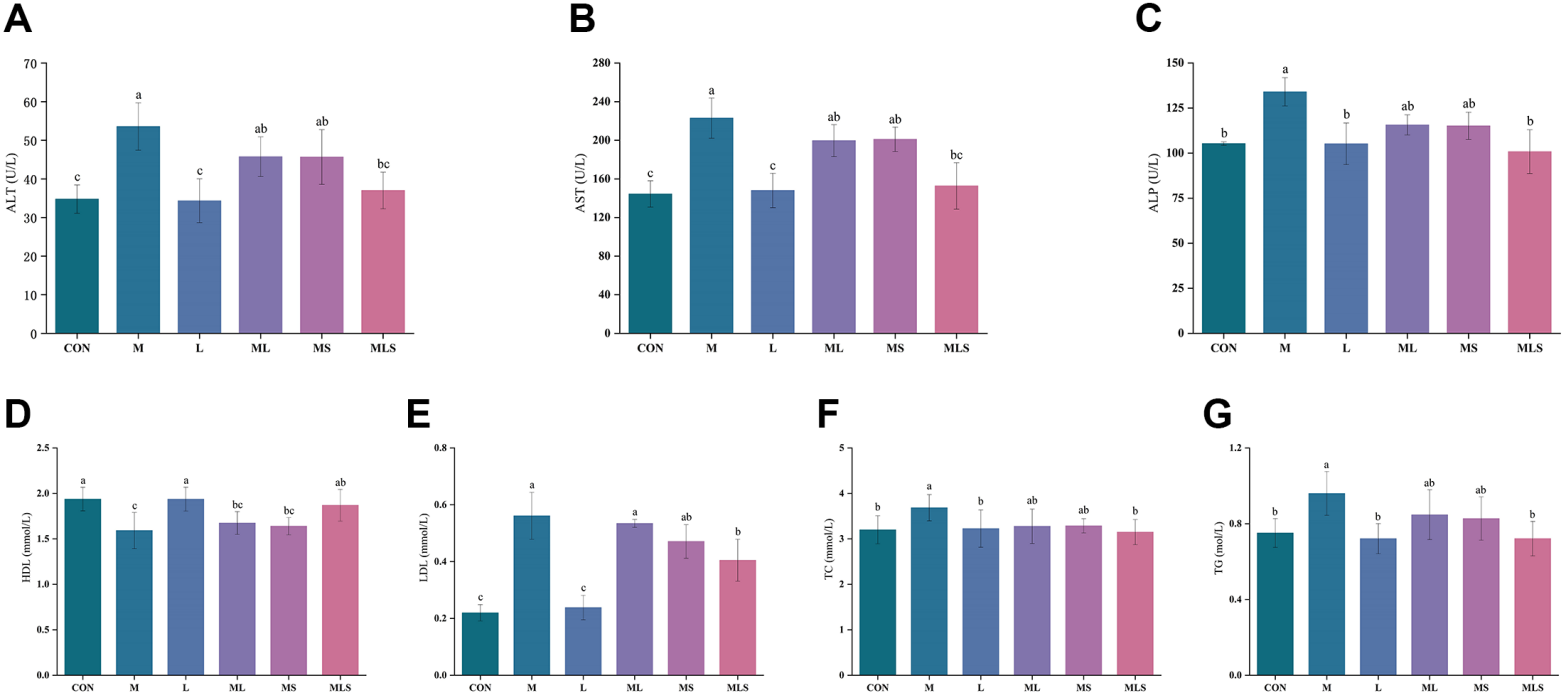
Serum biochemical indicators. (**A**) ALT, (**B**) AST, (**C**) ALP, (**D**) HDL, (**E**) LDL, (**F**) TC, (**G**)TG. Statistical significance among groups is denoted by differing letters (a, b, ab, c). Groups that do not share the same letter are significantly at *p* < 0.05.

The liver is an important organ for storing Cd, and liver damage can occur if Cd compounds are not excreted from the liver in a timely manner. Elevated serum levels of ALT, AST and ALP are regarded as important markers of impaired liver function. As shown in Fig. 3, Cd treatment significantly increased serum ALT, AST, and ALP levels in the M group compared with the CON group (*p* < 0.05). This indicates that Cd exposure caused severe liver injury in the mice. However, ALT, AST, and ALP levels were lower in the ML, MS, and MLS groups, than the M group. There was no significant difference between the ML, MS, and M groups, but a significant difference was observed between the MLS and M groups (*p* < 0.05).

The liver is an important organ involved in the metabolism of sugars and lipids. Elevated serum of TC, TG, and LDL levels and reduced serum HDL levels can indicate metabolic disorders in the liver. All of these abnormalities were observed in the M group, indicating that Cd exposure impaired lipid metabolism in the mouse liver. Se-enriched *L. plantarum* significantly improved the serum levels of TC, TG, LDL, and HDL compared with their levels in the M group (*p* < 0.05). Furthermore, the treatment administered to the ML and MS groups only restored TC to normal levels. In contrast, the treatment administered to the ML and MS groups resulted in reductions in TC, TG, LDL, and HDL levels, to levels that were not significantly different from those of the M group (*p* < 0.05).

### Histopathological Observations in the Liver

**Fig. 4:**
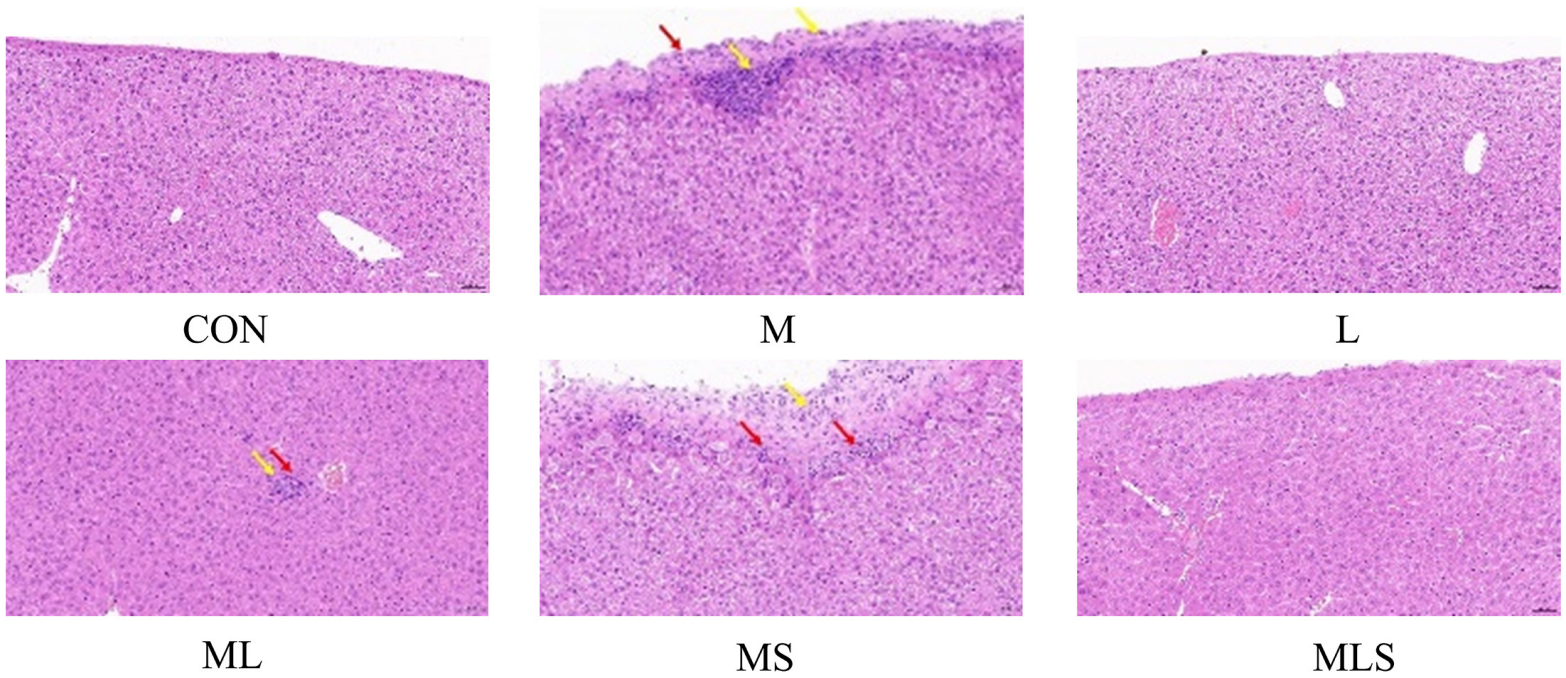
Effect of intervention on liver tissues of cadmium-exposed mice at 20.0x magnification.

Lipid metabolism disorders caused by Cd exposure can trigger liver inflammation. As shown in Fig. 4, a large number of hepatocytes with hydropic degeneration and cellular swelling were widely seen in the liver tissue of group M. Multiple lymphocyte and granulocyte infiltrations were seen in the tissue margins (yellow arrows), and localized thickening of the peritoneum and hyperplasia of the connective tissues were seen (red arrows).

Specially, the administration of Se-enriched *L. plantarum* significantly restored liver tissue structure to normal levels, similar to that in CON group (Fig. 4). No obvious inflammatory manifestations were seen in the MLS group. However, the treatment in the ML and MS groups still resulted in lymphocyte and granulocyte infiltration (yellow arrowheads), localized thickening of the peritoneum, and connective tissue hyperplasia (red arrowheads). These results suggested that treatment administered to the MLS group was more effective at protecting against Cd-induced liver injury.

### Oxidative Stress Indicators in Liver Tissues

**Fig. 5:**
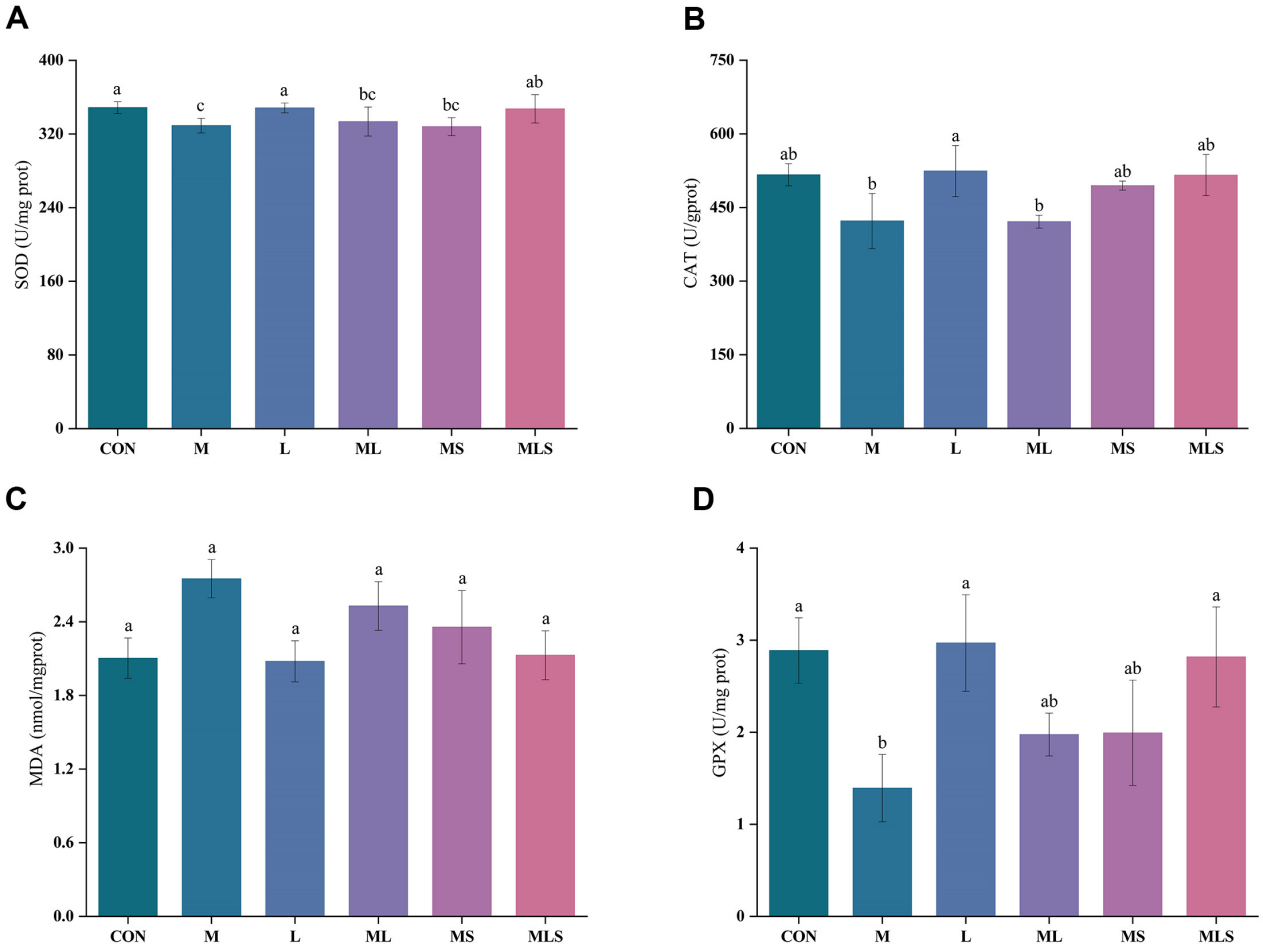
Effect of the intervention on the levels of oxidative stress in the livers of cadmium-exposed mice. (**A**) SOD, (**B**) CAT, (**C**) MDA, (**D**) GPX. Statistical significance among groups is denoted by differing letters (a, b, ab, c). Groups that do not share the same letter are significantly at *p* < 0.05.

Oxidative stress is a negative effect produced by free radicals in the body, and is an important factor leading to disease. The main pathogenic mechanism of Cd toxicity is that it can induce systemic oxidative stress [27]. A high level of MDA indicates oxidative stress, while high levels of SOD, CAT and GPX indicate higher levels of antioxidant activity to counteract reactive oxygen species and protect the liver from oxidative damage [28]. As shown in Fig. 5, the MDA content of the liver was significantly higher and the CAT, SOD and GPX contents were significantly lower after 4 weeks of Cd exposure. This indicated that the liver was in a state of oxidative stress. There was no significant difference in SOD, CAT, MDA and GPX between the M group and the ML group. The MS and MLS groups showed significant protection against Cd-induced changes in SOD, CAT, MDA, and GPX levels. The MLS group showed significant difference in these levels from those in the M group (*p* < 0.05).

### Changes in the Intestinal Microbiota

**Fig. 6:**
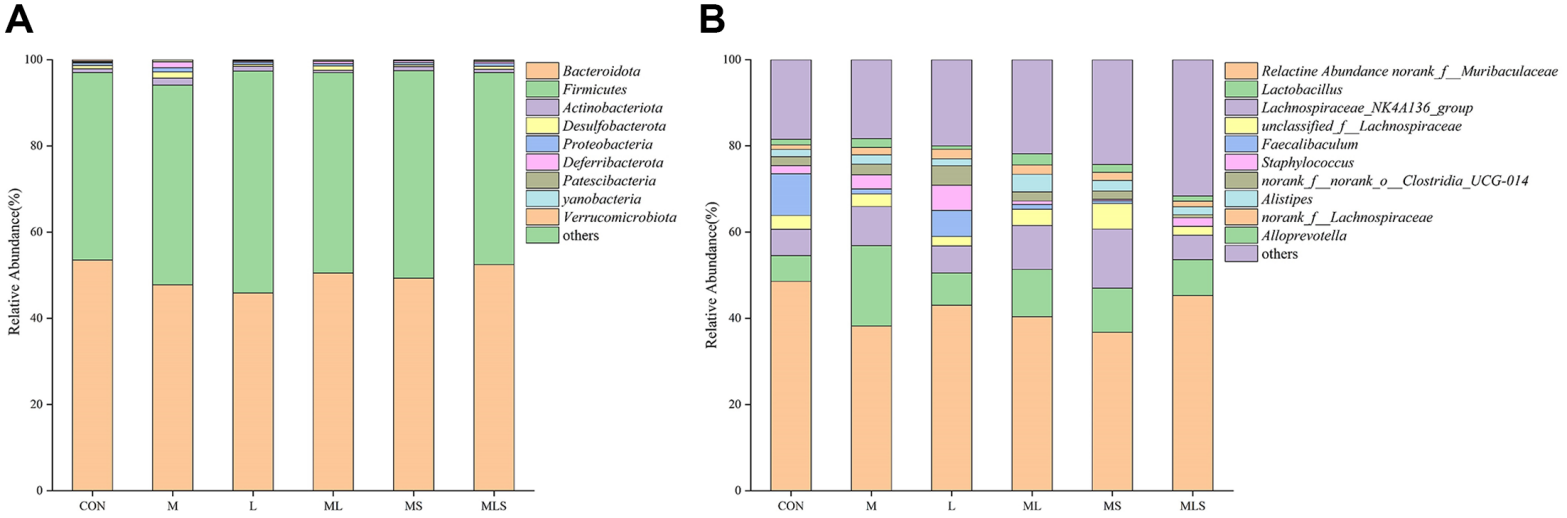
Effect of the intervention on gut microbiota diversity in cadmium-exposed mice. (**A**) Phylum level, (**B**) Genus level.

As shown in Table 1, Cd exposure resulted in significantly lower Ace and Shannon index values in the M group compared to the CON group, indicating lower α-diversity of the gut microorganisms (*p* < 0.05). There were no significant differences in Chao, coverage, Simpson, and Sobs index values among the groups. These findings were consistent with the fact that the species richness and evenness of the gut microbiota are less susceptible to environmental disturbances and stresses, as species with similar functions can fill ecological niches and maintain diversity when other species are compromised [29]. The ML, MS and MLS groups showed reduced Ace and Shannon diversity index values to near-normal levels (*p* < 0.05). At the phylum level, the ratio of Bacteroidota to Firmicutes is an important indicator of the energy harvesting capacity of gut microbiota, which is highly correlated with host intestinal health[30]. As shown in Fig. 6A, Cd exposure led to a decrease in the abundance of Bacteroidota and an increase in the abundance of Firmicutes compared with their abundance in the CON group. The treatment in the ML, MS, and MLS groups attenuated the changes induced by Cd exposure compared to the M group. The MLS group had the most effective treatment. As shown in Fig. 6B, at genus level, Cd exposure resulted in a decreased abundance of *norank_f_Muribaculaceae* and an increased abundance of *Lactobacillus* and *Lachnospiraceae_NK4A136*_group. *Norank_f_Muribaculaceae* reduces inflammation, inhibits harmful bacteria and oxidative stress, and ameliorates intestinal mucosal inflammation [31]. The increased abundance of *Lactobacillus* may be a result of Cd exposure making mice immunocompromised or mentally overstimulated, which usually leads to a decrease or increase of mycotoxins in the body. *Lachnospiraceae_NK4A136* _group is a conditionally pathogenic bacterium highly associated with intestinal flora dysbiosis [32]. The treatment in the MLS group reversed the above changes to a greater extent than in the ML and MS group. The treatment in the ML and MS groups succeeded in decreasing the abundance of *norank_f_Muribaculaceae* and *Lactobacillus*, but failed in elevating abundance of *Lachnospiraceae_NK4A136*_group.

**Fig. 7:**
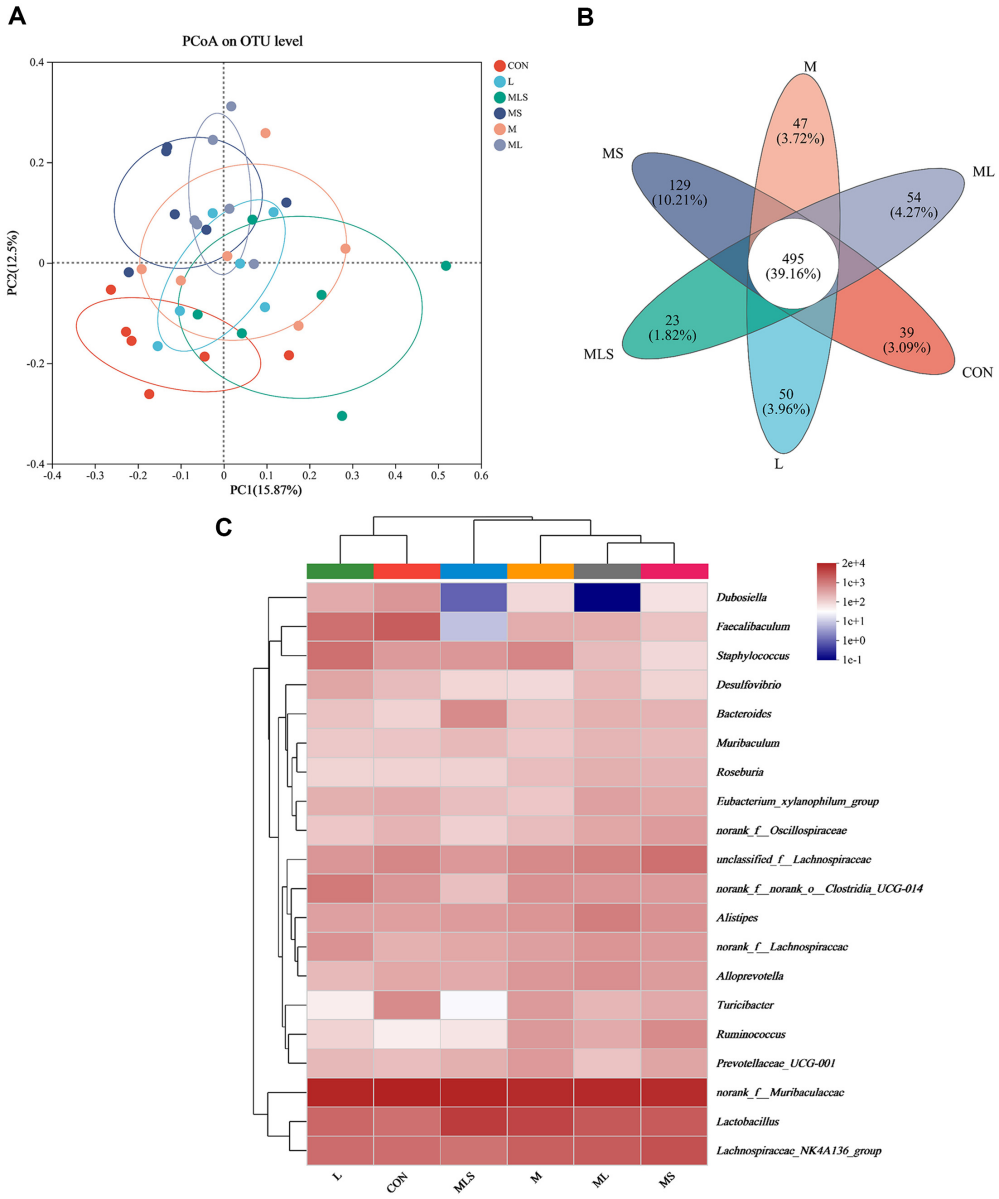
Effect of the intervention on the gut microbiota composition. (**A**) PCoA plot analysis, (**B**) Venn diagram analysis, (**C**) Community heatmap analysis on genus level.

To compare the β-diversity of the gut microbial in different groups, PCOA was performed based on the unweighted UniFrac distance algorithm. As shown in Fig. 7A, the main composition of the gut microbiota changed after Cd exposure, and the explanatory degree of the differences in sample composition between the main coordinate axes PC1 and PC2 was 15.87% and 12.5%, respectively, suggesting that Cd exposure had an influential effect on the composition of the gut microbiota. In this study, there were a total of 837 OTUs, of which the number of species common to the microbial composition of each group was 495 or 39.16% (Fig. 7B). To determine which species were responsible for the differences in microbial community composition, the 20 highest abundance genus-level microbial variations were analyzed (Fig. 7C). The results showed that the CON and L groups were grouped together, followed by the MLS group, and the ML and MS groups were farther apart, suggesting that Se-enriched *L. plantarum* was effective in transforming the gut microbiota.

### Relationship between the Intestinal Microbiota and Liver Injury

**Fig. 8:**
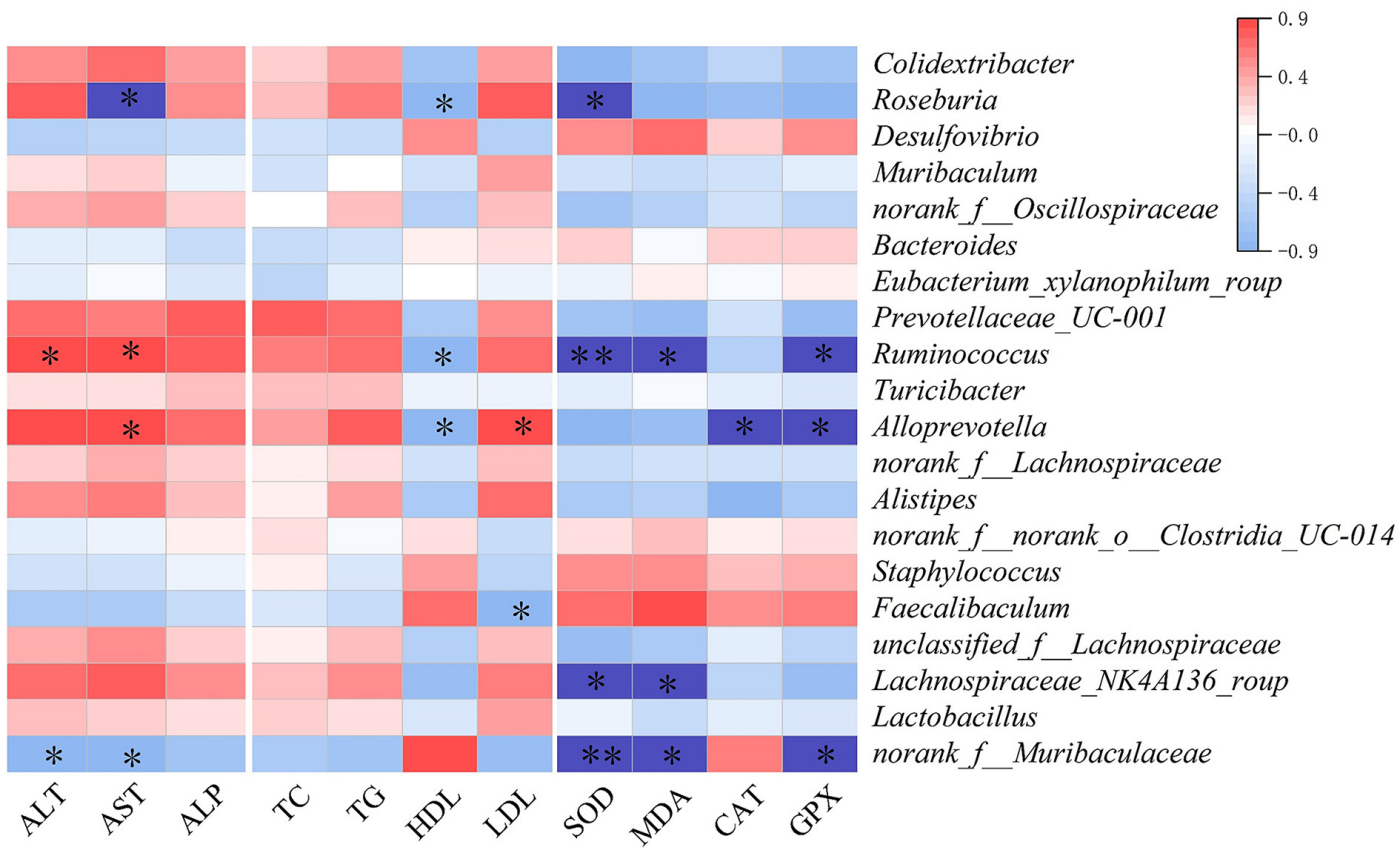
Heatmap of the Spearman correlation coefficient values between the top 20 enriched bacteria and liver function biomarkers. *Indicates the correlation is significant at *p* < 0.05 and ** significant at *p* < 0.01.

Fig. 8 showed the top 20 gut microorganisms at the genus level that were correlated with different indicators of Cd-induced liver injury. Cd-induced liver injury was consistent with the results of α-diversity analysis, and *Roseburia* abundance was negatively correlated with AST, HDL, and SOD (*p* < 0.05). *Roseburia*, a high butyric acid-producing bacterium, plays an important role in controlling intestinal inflammation. *Roseburia* has also been found to significantly improve liver inflammation, improve intestinal ecosystem and effectively prevent leaky gut [33]. The results showed that *Ruminococcus* was positively correlated with ALT and AST, and negatively correlated with HDL, SOD, and GPX levels (*p* < 0.05). It has been reported that *Ruminococcus* abundance is positively correlated with irritable bowel syndrome, as it can induce cells to secrete inflammatory cytokine, induce inflammatory bowel disease [34], and promote hepatic steatosis [35]. Therefore, the high efficiency of Se-enriched *L. plantarum* in mitigating Cd-induced liver injury can be explained by a significant increase in the abundance of *Ruminococcus* in the gut microbiota. In addition, *Alloprevotella* was positively associated with a high risk of irritable bowel syndrome. *Alloprevotella* was positively correlated with AST and LDL levels (*p* < 0.05), and negatively correlated with HDL, CAT and GPX levels (*p* < 0.05). *Lachnospiraceae_NK4A136*_group can produce butyric acid, and its abundance was negatively correlated with intestinal inflammation in proliferation process [36]. *Lachnospiraceae_NK4A136*_group was negatively correlated with SOD and MDA levels (*p* < 0.05). *Norank_f_Muribaculaceae* was negatively correlated with ALT, AST, SOD, MDA and GPX levels (*p* < 0.05). According to a previous report, *norank_f_Muribaculaceae* can regulate oxidative stress to affect the development of ulcerative colitis by the flora-gut-brain axis [37]. Therefore, modulating the gut microbiota may be a stratery for mitigating Cd-induced liver injury.

## Discussion

In this study, Se-enriched *L. plantarum* and SeNPs were prepared and isolated from reactions with *L. plantarum* and sodium selenite, respectively, and used as a basis for investigating the protective effects of different treatments on Cd-exposed mice. One study reported that Cd-exposed mice exhibited reduced activity, lethargy, and significant loss of final body weight [38]. Another study reported that CdCl2 exposure progressively reduced body weight gain in experimental animals, but combined treatment with different sources of Se significantly ameliorated weight loss induced by subchronic Cd exposure [39]. Consistent with the finding of previous studies, the results of the present study showed that Cd-exposed mice exhibited reduced activity, depression, and significant weight loss. No significant abnormal behaviors or signs were observed in the L and MLS groups compared to the CON group, indicating that the treatment of LS was effective in ameliorating Cd-induced adverse health conditions in mice.

The accumulation of Cd in the liver is an important indicator for assessing the degree of Cd toxicity. Some studies have shown that selenium can effectively reduce Cd toxicity by regulating selenoprotein expression [40]. *L. plantarum* can adsorb Cd ions before they are absorbed by the body and promote fecal excretion of Cd, thereby reducing damage. Consistent with the results of previous studies, *L. plantarum*, SeNPs and selenium-enriched *L. plantarum* all reduced Cd levels in the liver. However, compared to other studies, the present study found that Se-enriched *L. plantarum* was more effective than *L. plantarum* in ameliorating Cd toxicity. This may provide a new and effective method to treat Cd toxicity through biological methods.

Cd exposure can lead to hepatic lipid accumulation and liver inflammation in mice. A study in a mouse model found that Cd exposure led to hepatic lipid accumulation due to increased TG and TC levels in the serum and liver in mice [41]. Consistent with the findings of previous studies, Cd exposure in the present study increased the blood levels of ALT, AST, ALP, TC, TG, and LDL and decreased HDL levels. In response to these changes, it has been shown that Se-enriched *Lactobacillus* with the combined effect of organic selenium and *Bifidobacterium longum* significantly reduced serum ALT and AST activities and attenuated liver injury in rats [42]. Similarly, the treatment in the MLS group significantly improved the serum levels of ALT, AST, ALP, TC, TG, LDL, and HDL, and had a highly protective effect on the internal structure of liver tissue.

Liver damage is visually manifested by the destruction of liver tissue structure. Studies have shown that mice with Cd poisoning exhibit a loose arrangement of hepatocytes, cytoplasm filled with small vacuoles, inflammatory cell infiltration, and hepatocyte hemorrhage [43]. We observed no significant damage to liver tissue in the CON group. However, But group M exhibited cell swelling, multiple lymphocyte and granulocyte infiltration at the tissue edge, peritoneum thickening, and connective tissue hyperplasia. The results were consistent with previous tests, indicating that Cd can cause acute liver injury in mice. After the treatment, liver tissue injury was reduced in all groups. Compared to group M, mild infiltration of lymphocytes was evident in the liver tissue of groups ML and MS, but there were no significant inflammatory manifestations seen in the MLS group. This suggested that Se-enriched *L. plantarum* has the potential to be more efficient in ameliorating Cd-induced liver injury in mice.

Cd exposure induces oxidative stress, which leads to oxidative damage in different organs of the body. Oxidative stress can lead to activation of transcription factors through different signaling pathways, which in turn triggers pro-inflammatory cytokine production and apoptosis [44]. It has been shown that dietary supplementation with Se-enriched probiotics increased GPX and SOD activity and GSH content in mice, piglets and hens [45]. Similarly, Cd exposure reduced SOD, CAT and GPX levels and increased MDA levels in the present study, whereas different treatments for Cd-exposed mice all alleviated oxidative stress in the liver to some extent, with the MLS group having the most significant effect in comparison. Se-enriched *L. plantarum* increased SOD, CAT, and GPX levels by 5.5%, 22.1%, and 102.2%, respectively, and reduced MDA levels by 22.7%, all of which were restored to levels near those of the CON group. Indeed, it has also been shown that Se increases SOD levels and significantly decreases MDA levels [46]. The SOD-CAT system is the first line of defense against oxygen toxicity, and SOD can catalyze the disproportionation of superoxide to produce H_2_O_2_, which in turn is decomposed by CAT or GPX to H_2_O_2_. In the present study, the reduction of CAT and SOD activities induced by Cd exposure was observed, which may lead to the accumulation of H_2_O_2_, and consequently to the damage. Therefore, it is possible that Se-enriched *L. plantarum* may improve the activities of SOD and CAT by regulating the SOD-CAT system. However, further investigation is needed to determine the specific mechanism involved.

The intestinal microbiota has a complex composition, interacts well with intestinal epithelial cells, and plays an important role in the health and development of the host. Some studies have shown that the disturbance of the gut microbiota by Cd accumulation may affect various metabolic functions, leading to the development of various diseases [47]. *L. plantarum* CCFM8610 can protect the intestinal barrier and inhibit Cd uptake by attenuating oxidative stress [48]. Moreover, Se can prevent Cd-induced changes in Zn, Fe, and Cu levels by regulating transporter proteins, which has a preventive role in reducing Cd accumulation [49]. In this study, Cd exposure did dysregulate the intestinal microbiota. By analyzing the α-diversity and β-diversity of the intestinal flora of mice after treatment, it was found that Se-enriched *L. plantarum* was able to regulate the intestinal flora, inhibit the growth of pathogenic bacteria, promote the colonization of beneficial bacterial flora, and regulate intestinal microecology to alleviate the damage caused by Cd exposure to the organism.

Impaired liver function causes significant changes in the intestinal flora, which is an important component of the intestinal-liver axis and microecology. This can compromise the intestinal barrier function, allowing intestinal bacteria and their diverse metabolites to move into extraintestinal organs. This, in turn, activates the immune system and causes an abnormal immune response, ultimately leading to liver injury. It has been shown that monascin attenuates alcohol-induced oxidative damage in the liver by increasing the proportion of the flora of *Lactobacillus*, *Lachnospiraceae*_UCG-006, and *Coriobacteriales*, and decreasing the proportion of the flora of *Staphylococcus*, *Muribaculaceae*, *Desulfovibrionaceae*, and others to attenuate alcohol-induced oxidative damage in the liver [50]. In this study, by correlating some intestinal microorganisms with different indicators of Cd-induced liver injury, we found that the abundance of *Roseburia*, *Ruminococcus*, *Alloprevotella*, *Lachnospiraceae_NK4A136*_group, *norank_f_Muribaculaceae* significantly correlated with indicators of liver injury. Therefore, modulating Cd-exposed gut microbes may be a highly promising and safe treatment.

In summary, the results of this study indicated that Se-enriched *L. plantarum* effectively reduced Cd accumulation in the liver, had a high mitigating effect on Cd intoxication in mice, attenuated Cd-induced hepatic injury and, oxidative stress, and restored the intestinal microflora to an approximately normal level. Changes in the abundance of *Ruminococcus*, *Alloprevotella*, and *norank_f_Muribaculaceae* in the intestine may play an important role in the occurrence and prevention of Cd-induced liver injury. This may also be the reason why Se-enriched *L. plantarum* was a more effective treatment than *L. plantarum* and SeNPs; however more in-depth studies are needed to confirm this.

## Figures and Tables

**Table 1:** Effects of the interventions on α- diversity in cadmium-exposed.